# A Novel *CCM2* Gene Mutation Associated with Familial Cerebral Cavernous Malformation

**DOI:** 10.3389/fnagi.2016.00220

**Published:** 2016-09-21

**Authors:** Wen-Qing Huang, Cong-Xia Lu, Ya Zhang, Ke-Hui Yi, Liang-Liang Cai, Ming-Li Li, Han Wang, Qing Lin, Chi-Meng Tzeng

**Affiliations:** ^1^Translational Medicine Research Center, School of Pharmaceutical Sciences, Xiamen UniversityXiamen, China; ^2^Key Laboratory for Cancer T-Cell Theranostics and Clinical TranslationXiamen, China; ^3^Department of Neurology, The First Affiliated Hospital of Xiamen UniversityXiamen, China; ^4^The First Clinical College of Fujian Medical UniversityFuzhou, China; ^5^INNOVA Cell: TDx/Clinics and TRANSLATE Health GroupYangzhou, China

**Keywords:** familial cerebral cavernous malformations (FCCM), susceptibility-weighted imaging (SWI), *CCM2*, mutation, pathogenesis, biomarker

## Abstract

**Background:** Cerebral cavernous malformations (CCMs) are common vascular malformations that predominantly arise in the central nervous system and are mainly characterized by enlarged vascular cavities without intervening brain parenchyma. Familial CCMs (FCCMs) is inherited in an autosomal dominant pattern with incomplete penetrance and variable symptoms.

**Methods:** Mutations of three pathogenic genes, *CCM1, CCM2*, and *CCM3*, were investigated by direct DNA sequencing in a Chinese family with multiple CCM lesions.

**Results:** Four heterozygous variants in the *CCM2* gene, including one deletion (c.95delC), a missense mutation (c.358G>A, p.V120I), one silent mutation (c.915G>A, p.T305T), and a substitution (c. ^*^1452 T>C), were identified in the subjects with multiple CCM lesions, but not in a healthy sibling. Among these variants, the c.95delC deletion is a novel mutation which is expected to cause a premature termination codon. It is predicted to produce a truncated *CCM2* protein lacking the PTB and C-terminal domains, thus disrupting the molecular functions of *CCM2*.

**Conclusions:** The novel truncating mutation in the *CCM2* gene, c.95delC, may be responsible for multiple CCM lesions in a part of FCCM. In addition, it may represent a potential genetic biomarker for early diagnosis of FCCM.

## Introduction

Cerebral cavernous malformations (CCMs) are common vascular defects with a prevalence of 1 in every 200–250 individuals. They mostly occur in the central nervous system (e.g., the forebrain, brainstem, cerebellum, spinal cord, cranial nerves, and cerebral ventricles, Gomori et al., 1986; Rigamonti et al., 1987). Histologically, CCMs are characterized by abnormally enlarged capillary-like vascular channels lined by a single layer of endothelium without intervening brain parenchyma (Gomori et al., 1986; Rigamonti et al., 1987). As one of the most common types of vascular malformations found in the brain, CCMs represent up to 15% of all CNS vascular malformations (Cavalcanti et al., 2012). They affect up to 0.5% of the global population, accounting for approximately 24 million people worldwide (Rigamonti et al., 1988; Otten et al., 1989; Cavalcanti et al., 2012), and their observed prevalence will increase with the widespread application of magnetic resonance imaging (MRI), and especially susceptibility-weighted imaging (SWI), in clinical diagnosis (de Souza et al., 2008).

Clinical symptoms of CCMs usually appear between 20 and 50 years of age (with an average age of onset of approximately 30 years), but they can also arise in early infancy or old age (Labauge et al., 2007; Cavalcanti et al., 2012). The most common manifestations include seizures, recurrent headaches, hemorrhagic stroke, epileptic attacks, and focal neurological deficits, but some CCMs can also be relatively asymptomatic (Labauge et al., 1998, 2007). The percentage of CCMs patients who remain symptom-free throughout their lives has been estimated to be as high as 40% (Labauge et al., 2007; Mondejar et al., 2014).

CCMs can present as either a sporadic form, usually with a single lesion, or familial form, mostly with multiple lesions (Rigamonti et al., 1988). The proportion of Familial CCMs (FCCM) has been estimated to be as high as 50% in Hispanic-American patients and close to 10–40% in Caucasian patients (Rigamonti et al., 1988; Labauge et al., 2007). The prevalence of FCCM has not been investigated in the Chinese population. FCCM are inherited in an autosomal dominant fashion with incomplete penetrance and variable expressivity (Bicknell et al., 1978). To date, three causative genes have been identified in the etiology of FCCM (Cavalcanti et al., 2012). They are *CCM1* (*KRIT1*), *CCM2*, and *CCM3* (*PDCD10*), which encode the proteins krev/rap1 interacting trapped 1 (Krit1), malcavernin, and programmed cell death 10 (PDCD10), respectively (Labauge et al., 2007; Cavalcanti et al., 2012). These three proteins form a heterotrimeric complex that is critical for different molecular pathways, including angiogenesis and normal vascular morphogenesis in the brain (Zawistowski et al., 2005; Voss et al., 2007; Pagenstecher et al., 2009; Faurobert and Albiges-Rizo, 2010; Li et al., 2010; Stockton et al., 2010; Cavalcanti et al., 2012). The loss of these functions of CCM proteins resulting from mutations in CCM genes leads to CCMs (Stahl et al., 2008; Chan et al., 2011).

Here, we report the clinical and neuroradiological features of a Chinese family with multiple CCMs, along with a genetic analysis of the *CCM1/CCM2/CCM3* genes in three family members through a genomic DNA-targeted sequencing method. We identify four mutations in *CCM2*, among which one mutation (c.95 delC) in *CCM2* is novel and may contribute to the pathogenesis of a part of FCCM.

## Subjects and methods

### Subjects

**Figure 1:**
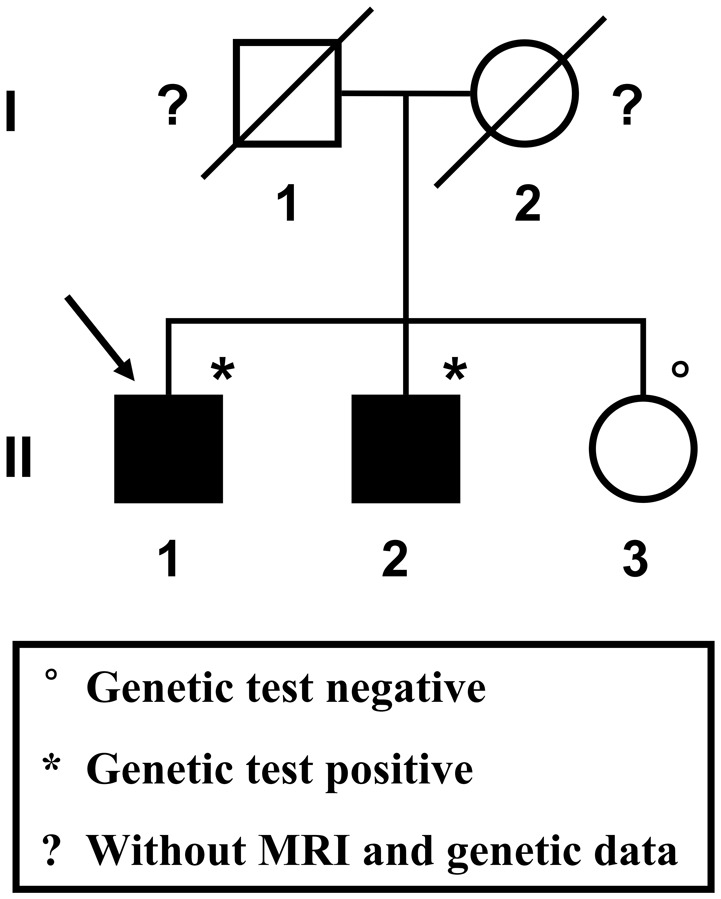
**Pedigree of the studied Chinese family**. The proband is indicated by the arrow. Squares represent males; circles represent females. Black-filled symbols indicate a member showing multiple cerebral cavernous malformations upon SWI-MRI of the brain. A diagonal line through the symbol represents a deceased person.

**Figure 2:**
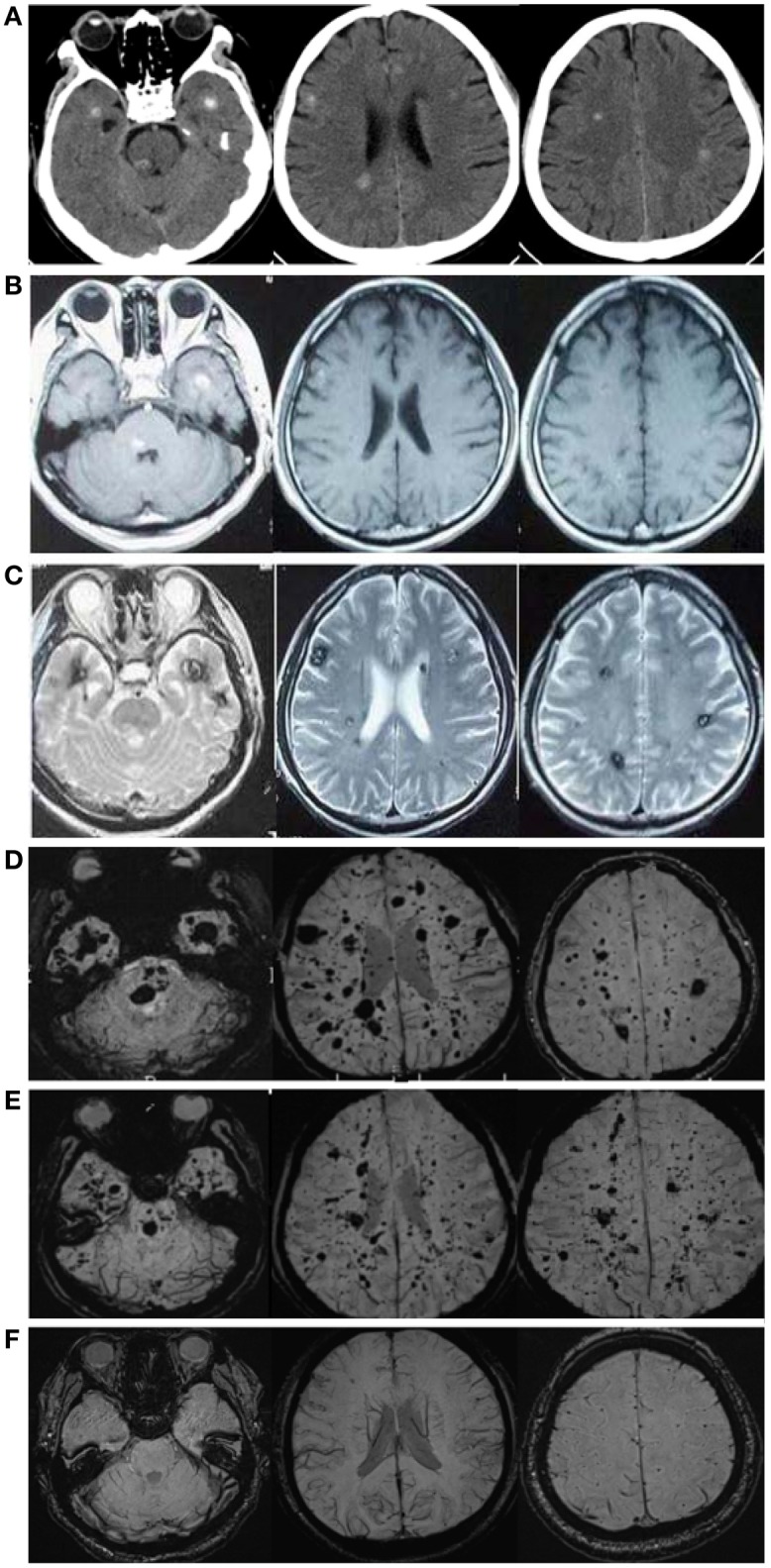
**CCM lesions diagnosed through CT, GE-and SWI-MRI. (A)** CT of the 57-year-old proband, II-1. A plain CT scan (brain window images) showed multiple calcification spots scattered on the tegmentum of the pons, temporal lobes and periventricular area. **(B)** Axial T1-weighted gradient-echo (GE) MR imaging of the proband, II-1. Axial T1-weighted GE images demonstrated hyper-intensity of hemorrhage lesions. **(C)** Axial T2-weighted gradient-echo (GE) MR imaging of the proband, II-1. T2-weighted GE images showed a “popcorn” appearance surrounded by a dark rim of hemosiderin. **(D)** SWI-MR imaging of the proband, II-1. SWI revealed thickly dotted CCMs distributed throughout the cerebral cortex in the brain of the proband. There are dozens of lesions on the cerebrum, cerebellum, thalamus, and brain stem. The diameter of the lesions ranges from a few millimeters to several centimeters. **(E)** SWI-MR imaging of the proband's elder brother, II-2. II-2 showed a similar phenomenon, with multiple CCM lesions observed upon SWI-MRI of the brain. **(F)** SWI-MR imaging of the proband's younger sister, II-3. II-3 did not show any CCM lesions upon SWI-MRI of the brain.

A 57-year-old male patient (Figure 1 II-1), the proband, arrived at the department of neurology, the First Affiliated Hospital of Xiamen University, Xiamen, Fujian, China, showing symptoms of diplopia and hemidysesthesia only on the left. He denied any fever, headache, vomiting, vertigo, hypertension, or trauma as well as any other prior history (especially disorders in central nervous system). A physical exam revealed paralysis of the sixth nerve on the right side, but the other cranial nerves appeared normal. No other neurological sign was observed during his presentation and clinical observation. His hematological and biochemical data from blood and cerebrospinal fluid tests were normal. Serological tests for various agents, including parasite antibodies were all negative. Non-contrast computed tomography revealed multiple high-intensity patchy calcifications or bleeding distributed around the cortical and subcortical regions of the cerebral hemispheres, cerebellum and brain stem (Figure 2A). Surprisingly, many additional thick CCMs distributed throughout the brain (including the cerebrum, cerebellum, thalamus, and brain stem) were detected in susceptibility-weighted images (SWIs, Figure 2D). However, they could not be detected by T1 or T2-weighted gradient echo (GRE) sequences (Figures 2B,C). The diameters of the lesions ranged from 0.5 mm to 3 cm, averaging 0.9 cm. The patient's elder brother (Figure 1 II-2, aged 63 years) and younger sister (Figure 1 II-3, aged 53 years) were asymptomatic. Both siblings also denied trauma, prior history, or other special medication history. They were also informed that they should receive SWI scans. Although the proband's elder brother (II-2) did not presented much more and severer CCM lesions on the cerebellum, cerebrum, thalamus, and brain stem than the proband, he also showed a similar phenomenon, with multiple CCM lesions in sections of the sellar region and parietal lobe upon sagittal and coronal SWI imaging of the brain (Figure 2E). The proband's sister (II-3) did not show any CCMs in the SWI scan of the brain (Figure 2F). The proband's parents died several years ago. Therefore, their MRI data were not available. Whole blood samples were obtained from patient II-1 and his siblings, subjects II-2 and II-3. This study was approved by the ethics committee of Xiamen University. All study subjects provided written informed consent.

### Genetic analysis

Genomic DNA was extracted from whole blood of the subjects using the MagCore Genomic DNA Whole Blood Kit. All of the coding exons of the *CCM1* (GenBank: NG_012964.1, including 20 exons), *CCM2* (GenBank: NG_016295.1, including 10 exons), and *CCM3* (GenBank: NG_008158.1, including 9 exons) genes were PCR amplified using a specific subset of primer pairs (Supplemental Table 1). Then, the purified PCR products were directly sequenced with a 3730 automatic sequencer (Applied Biosystems, USA). The DNA sequences were analyzed by the Sequencing Analysis Version 5.2 software package (Applied Biosystems). Sequence alignment was performed with DNAMAN software. The detected variants were described according to the recommendations of the Human Genome Variation Society, taking the A of the ATG translation initiation codon as +1 at the cDNA level. The variants were considered novel when they were not reported in previous publications or in the following public databases: (1) CCM mutation database (http://www.angiomaalliance.org/pages.aspx?content=345&id=289); (2) National Center for Biotechnology Information (NCBI) dbSNP (http://www.ncbi.nlm.nih.gov/snp/); (3) Exome Variant Server for Exome Sequencing Project from the National Heart, Lung, and Blood Institute (http://evs.gs.washington.edu/EVS/); and (4) The Human Gene Mutation Database (HGMD) (http://www.hgmd.org/). The effects of these variants were evaluated with several types of predictive software, including (1) MutationTaster (http://www.mutationtaster.org/); (2) Mutalyzer (https://mutalyzer.nl/); (3) SplicePort (http://spliceport.cbcb.umd.edu), (4) Alternative Splice Site Predictor (ASSP) (http://wangcomputing.com/assp/index.html), and (5) Human Splicing Finder (http://www.umd.be/HSF/). Finally, the variants associated with the pathogenesis of FCCM were further examined in 200 unrelated ethnically matched normal controls.

## Results

**Figure 3:**
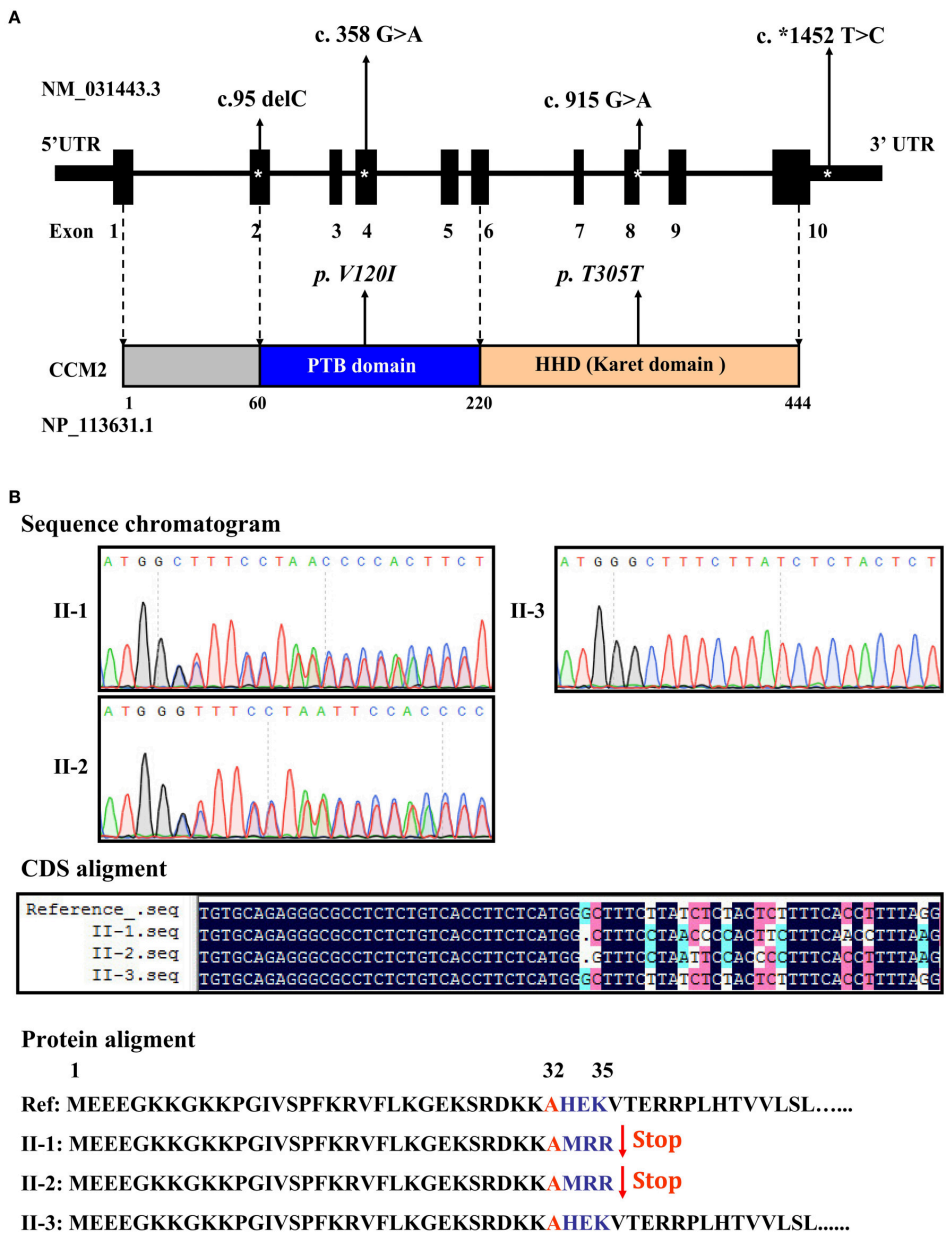
**Distribution and function of mutations in the ***CCM2*** gene. (A)** Schematic representation of four *CCM2* gene mutations identified in the domains of *CCM2*-coding protein in the studied Chinese family with FCCM. **(B)** A novel frameshift mutation in exon 2 of the *CCM2* gene. This deletion (c.95 delC), located in exon 2, was heterozygous in both siblings with CCMs. It resulted in a stop codon in the 36th original codon and produced a 35-amino acid, truncated form of CCM2, without the PTB domain and all of the C-terminal domains.

Targeted sequencing of the coding exons and intronic boundaries of three CCM family genes revealed 4 variants in the *CCM2* gene in both the proband (II-1) and his brother (II-2), but not in his healthy sister (II-3). No variants were identified in the *CCM1* or *CCM3* genes in any of the siblings. The *CCM2* variant 2, NM_031443.3, is selected as the reference sequence of those variants (Stahl et al., 2008). The four identified variants were heterozygous in both siblings and are summarized in Table 1, including one deletion in (c.95delC) exon 2, a missense mutation (c.358G>A, NP_113631.1: p.V120I) in exon 4, one silent mutation (c.915G>A, NP_113631.1: p.T305T) in exon 8, and a substitution (c.^*^1452 T>C) in exon 10 (Figure 3A). Among these variants, only the c.95delC deletion was a novel mutation that had not been reported in the NCBI dbSNP, HGMD, and CCM mutation databases or the previous literature. Moreover, direct sequence analysis of 200 healthy controls confirmed that it was not a polymorphism but a mutation. Additionally, pathogenicity analysis revealed that the novel deletion (c.95delC) in the *CCM2* gene was a frameshift mutation, which could result in premature termination at the 35th codon and cause a major truncation corresponding to the last 409 amino acids at the C-terminus of the full CCM2 protein (Figure 3B). Additionally, together with MutationTaster and SplicePort, HSF predicted that this mutation might disrupt the splice site and lead to a potential splicing alteration. According to the applied *in silico* analysis tools, three other variants were also predicted to alter donor splice sites, probably affecting splicing. These variants were considered as three single nucleotide polymorphisms (SNPs), rs11552377 (c.358G>A), rs2289367 (c.915G>A), and rs7804 (c.^*^1452 T>C), which could be found in the NCBI dbSNP database. They appeared to occur in European, European American, African American, and Asian Chinese populations. Among the variants identified in the family examined in the present study, the c.^*^1452 T>C (rs7804) substitution was the only mutation located in the 3′ UTR region (Figure 3A). miRNA binding site prediction analysis did not indicate that its neighboring region might act as a potential miRNA binding site, suggesting that the c.^*^1452 T>C variant could not contribute to the pathogenesis of FCCM through the regulation of *CCM2* mRNA expression.

**Table 1:** **Mutations identified in siblings with CCMs (II-1 and II-2)**.

	**Region**	**Nucleotide change (cDNA)**	**Amino acid change**	**Function**	**Domain**	**Mutation**	**Type**
CCM2	Exon 2	c.95 del C	p. A32A	Delection, Frameshift	N-terminal domain	Novel	Heterozygous
CCM2	Exon 4	c.358 G>A	p. V120I	Missense	PTB domain	Known (rs11552377)	Heterozygous
CCM2	Exon 8	c.915 G>A	p. T305T	Silent	C-terminal Karet domain	Known (rs2289367)	Heterozygous
CCM2	3′UTR (Exon 10)	c.*1452 T>C	Null	Unknown	Null	Known rs7804)	Heterozygous

*Genbank accession NM_031443.3 (CCM2 variant 2). The first nucleotide of ATG translation codon is considered nt +1. PTB, Phosphotyrosine-binding domain*.

## Discussion

Familial cerebral cavernous malformations (FCCM), which are commonly characterized by the presence of multiple lesions upon cerebral computed tomography/MR imaging, are a rare autosomal-dominant inherited disorder with incomplete penetrance and wide phenotypic variability (Bicknell et al., 1978; Labauge et al., 2007). FCCM are more likely to grow and form new lesions and to present hemorrhages and neurological signs than sporadic CCMs (Clatterbuck et al., 2000; Maiuri et al., 2006; Labauge et al., 2007). Therefore, the identification of a symptomatic proband with multiple CCM lesions prompted us to perform both presymptomatic MRI screening and genetic testing in the asymptomatic relatives to clarify this potential case of familial CCMs. In the present study, we did not find any mutations in CCM genes in the asymptomatic sibling without CCM lesions, but we did identify four variants in the *CCM2* gene in two siblings with multiple CCM lesions, including one novel frameshift mutation and three known polymorphisms. These findings therefore confirmed this proband as a familial CCMs case, rather than a sporadic CCMs case.

The pathogenesis of FCCM is mainly attributed to defects in the *CCM1, CCM2*, and *CCM3* genes (Stahl et al., 2008; Cavalcanti et al., 2012; Draheim et al., 2014). More than 200 distinct germline mutations in those three genes have been reported to date (Cavalcanti et al., 2012; Zhu et al., 2014); however, they account for only 70–80% of all FCCM cases (Denier et al., 2006; Cavalcanti et al., 2012). Here, the *CCM2* gene mutations identified in two siblings may account for the etiology of a part of FCCM cases in China. *CCM2* encodes a 444-amino acid protein, also known as malcavernin, that contains a phosphotyrosine-binding (PTB) domain (encoded approximately by residues from 60 to 220) at its N-terminus and a harmonin homology domain (HHD) (encoded by residues approximately from amino acids 220 to 444) at its C-terminus (Fisher et al., 2013, 2015). It has been shown that CCM2 can bind to CCM1 (Krit1) and CCM3 (PDCD10) through its PTB domain, thereby regulating cell-cell junctions, cell-extracellular matrix adhesion and the proliferation and migration of cells (Zawistowski et al., 2005; Zhang et al., 2007; Faurobert and Albiges-Rizo, 2010; Li et al., 2010; Gingras et al., 2012). CCM2 has also been found to act as scaffold for the actin cytoskeleton machinery through simultaneously binding Rac1 and MEKK3 in the p38 MAPK pathway to regulate cytoskeleton reorganization (Uhlik et al., 2003; Zawistowski et al., 2005). Additionally, Liraz Harel et al. have shown that CCM2 interacts with TrkA via its PTB domain and mediates TrkA-induced cell death through its Karet domain in neuroblastoma or medulloblastoma (Harel et al., 2009). These findings indicate the crucial roles of *CCM2* in the pathogenesis of vascular defects in FCCM.

The c.95 delC mutation identified in the present study was expected to generate a stop codon leading to the predicted formation of a 35-amino acid truncated protein lacking the original domains. This truncating mutation may disrupt all of the functions of CCM2 in the maintenance of vascular integrity and remodeling, thus triggering the onset of lesions. Why did the two siblings with multiple CCM lesions not show severe clinical symptoms, such as seizures and hemorrhage? The reason may be that they were heterozygous for both mutations and retained a portion of CCM function. Our hypothesis is supported by a study that has revealed that loss-of-function (LOF) mutation in *CCM2*, c.95 delC, cause endothelial cell dysfunction and loss of vascular integrity in animal models (Boulday et al., 2009; Chan et al., 2011). In line with our findings, previous studies in many of FCCM cases have identified more than 40 LOF mutations within the *CCM2* gene, which were either missense mutations or frameshift deletions, that destroyed the scaffold function of the protein or directly generated a truncated protein without any functional domains (Liquori et al., 2003; Stahl et al., 2008; Mondejar et al., 2014). These results indicate that LOF mutations of *CCM2* could be an important cause of FCCM.

Bioinformatic analysis showed that the c.915G>A substitution in the Karet domain and c.^*^1452 T>C substitution in the 3′UTR occurred within splicing sites, as observed for the above variant, c.95delC. Although neither mutation altered the amino acid sequence of CCM2 or potential miRNA binding sites, they may contribute to the pathogenesis of FCCM by altering splicing. Because RNA analysis has not been performed in this present study, it will be necessary to evaluate the effect of putative splicing mutations on the expression and function of CCM2 in the future.

Furthermore, multiple alignment analysis revealed that as same as the amino acids residues, 32Ala and 305Thr, 120Val that is located in the PTB domain and is changed by the c.358G>A variant is highly conserved among mammal (Homo sapiens, Mus musculus, Rattus norvegicus, Macaca mulatta, Bos taurus, Pan troglodytes, Canis lupus familiaris, Sus scrofa). Compared with rs2289367 (c.915G>A) and rs7804 (c.^*^1452 T>C), the minor allele frequency (MAF) of rs11552377 (c.358G>A) is relative low, observed in 1000 Genomes (MAF = 0.1080) and ExAC (MAF = 0.1461) databases. Additionally, the frequency of the c.358G>A variant in Han-Chinese (MAF = 0.07) is much lower than that in European (MAF = 0.18), but is a little higher than that in African American (MAF = 0.03). The high MAF of c.358G>A (rs11552377) in general population suggested it as a benign polymorphism, rather a disease-causing mutation. Together with Mutation Taster, both SIFT (Sorting Intolerant From Tolerant) and Polyphen-2 (Polymorphism Phenotyping v2) prediction of functional effects of c.358G>A (rs11552377) further supported this opinion. Therefore, we think that the novel truncating mutation, c.95delC, could be the driven factor for multiple CCM lesions in the cases. The c.358G>A variant changing the amino acid residue could not contribute to the pathogenesis of FCCM through disrupting the structure of PTB domain. Given that those variants were just identified in limited cases in this study, we thought that it was necessary to verify them in more FCCM cases, moreover confirm its significance in the pathogenesis of FCCM.

Confusingly, one of the proband's siblings did not show any manifestations but presented similar multiple CCM lesions and a mutation pattern consistent with the affected proband. The causes of this variability are unknown. We suggest that it was likely associated with the incomplete clinical penetrance of FCCM. The reason why the elder brother with multiple CCM lesions showed asymptomatic may be the insufficiency of lesions on several functional regions of the brain (e.g., cerebellum, cerebrum, thalamus, and brain stem), especially on the junction of the medulla oblongata and the pons that were related with the diplopia and paralysis of those nerves. Additionally, other genetic factors (such as the putative *CCM4* gene), environmental factors (causing different epigenetic regulation) or lifestyle could also contribute to this difference in clinical penetrance between these two siblings. Therefore, we think that *CCM2* variants (e.g., the c. 95delC mutation) could be the driven factors for multiple CCM lesions in both affected brothers. The different clinical manifestations of two brothers are ascribed to the different location and severity of CCM lesions, which may be the resulted from individual differences in other unknown gene variants or lifestyle associated epigenetics. To address such incomplete clinical penetrance or phenotype variability among FCCM cases, further studies are required to explore genotype-phenotype correlations, particularly in relation to the nature of the mutated gene in animal models.

## Conclusion

In conclusion, we report a case of FCCM and its associated symptoms and genotypes in a Chinese family. Among the four identified variants in *CCM2* gene, the c. 95delC mutation has not been previously identified, either in China or elsewhere, and it may represent a potential biomarker for FCCM cases. Moreover, this novel mutation is expected to lead to a frameshift mutation and produce a truncated form of CCM2 without the PTB domain and all of the C-terminal domains, thus disrupting the molecular functions of CCM2. Thus, it may contribute to the pathogenesis of multiple CCM lesions in a part of FCCM cases. We believe that it is necessary for clinicians and neuroscientists to identify unaffected carriers of such mutations and asymptomatic individuals with multiple CCM lesions in cases of FCCM as early as possible, in addition to understanding the etiology of FCCM through the use of neuroradiology screening and genetic counseling.

## Author contributions

QL and CL performed the clinical evaluation, neuropsychological and psychiatric assessment and MRI examination of patients. WH and KY carried out the blood sample and clinical information collection. WH and LC carried out all genetic test, screening and validation. YZ and ML participated in the validation of novel mutations and the sequence alignment. WH polished the figures and tables. CT and QL conceived of the study and drafted manuscript. All authors read and approved the final manuscript.

### Conflict of interest statement

The authors declare that the research was conducted in the absence of any commercial or financial relationships that could be construed as a potential conflict of interest.

## Abbreviations

FCCM: familial cerebral cavernous malformations
LOF: loss-of-function
MRI: magnetic resonance imaging
PTB: phosphotyrosine-binding
SNP: single nucleotide polymorphisms
SWI: susceptibility-weighted imaging.
